# A Highly Specific Biomarker for Early Diagnosis and Treatment of Neovascular Age-Related Macular Degeneration

**Published:** 2010-01

**Authors:** Khalil Ghasemi Falavarjani

**Affiliations:** Rassoul Akram Hospital, Iran University of Medical Sciences, Tehran, Iran

Age-related macular degeneration (AMD) is the most common cause of legal blindness among the elderly in developed countries. The prevalence of advanced forms of AMD is increasing; it is expected to rise by 50% by the year 2020 in the United States. Early identification of AMD susceptibility and implementation of preventive measures are important management strategies. Antibodies against vascular endothelial growth factor (VEGF) have become the treatment of choice in patients with choroidal neovascularization (CNV); however, substantial improvement of vision occurs only in approximately one-third of patients. Early diagnosis and treatment of CNV may increase the success rate of treatment.

Standard diagnostic modalities including fundus angiography and optical coherence tomography can detect CNV in clinical stages when vision is affected. In a novel approach, Takeda and coworkers evaluated the role of an eosinophil/mast cell chemokine receptor, CCR3, in CNV. They studied CCR3 expression among patients with AMD and previously untreated surgically excised CNV membranes, donor eyes of age-matched non-AMD controls, atrophic AMD, uveal melanoma, and surgically excised epiretinal membranes. Additionally, in vivo CCR3 bioimaging was performed in transgenic mice with spontaneous CNV using intravenous quantum-dot labeled CCR3 antibody fragments followed by fundus fluorescent imaging which demonstrated hyperfluorescent signals in regions of the fundus that were silent on standard fluorescein angiography. These hyperfluorescent dots were subsequently confirmed to be CNVs using histologic examination. The investigators showed that CCR3 was specifically expressed in the endothelial cells of all human CNV membranes but not in other conditions. Interestingly, they found that CCR3 neutralization was superior to VEGF blockade in suppressing laser-induced CNV in mice. Considering emerging safety concerns about continual blockade of VEGF, which is constitutively expressed in the normal adult human retina and needed for retinal health, a treatment strategy based on more specific targeting of CNV would be desirable.

These findings suggest that CCR3 targeting may be a viable strategy for early detection and treatment of CNV, and might be superior to the current standard of care. CCR3 bioimaging would probably be most useful in eyes at high risk for CNV such as multiple large drusen or sound fellow eyes of patients with clinically evident CNV; it can also serve as a useful tool for differentiating lesions mimicking CNV.
